# Supplementary material for: Incidence, recurring admissions and mortality of severe bacterial infections and sepsis over a 22-year period in the population-based HUNT study
Source: PLoS One. 2022 Jul 12;17(7):e0271263. doi: 10.1371/journal.pone.0271263 (PMC9275692; doi:10.1371/journal.pone.0271263)
Supplement: S1 Table — (PDF) [file pone.0271263.s001.pdf]

ICD-10		ICD-9	
**Pneumonia**			
J13	Pneumonia due to Streptococcus pneumoniae	481	Pneumococcal pneumonia
J14	Pneumonia due to Haemophilus influenzae	482.2	Pneumonia due to Hemophilus influenzae
J15.0	Pneumonia due to Klebsiella pneumoniae	482.0	Pneumonia due to Klebsiella pneumoniae
J15.1	Pneumonia due to Pseudomonas	482.1	Pneumonia due to Pseudomonas
J15.2	Pneumonia due to Staphylococcus	482.4	Pneumonia due to Staphylococcus
J15.3	Pneumonia due to Streptococcus, group B	482.3	Pneumonia due to Streptococcus
J15.4	Pneumonia due to other streptococci		
J15.5	Pneumonia due to Escherichia coli	482.8	Pneumonia due to other specified organism
J15.6	Pneumonia due to other gram negative bacteria		
J15.7	Pneumonia due to Mycoplasma pneumoniae	483.0	Pneumonia due to other specified organism
J15.8	Other bacterial pneumonia		
J15.9	Bacterial pneumonia, unspecified	482.9	Bacterial pneumonia, unspecified
J16	Chlamydial pneumonia		
J16.8	Pneumonia due to other specified infectious organisms		
J18.0	Bronchopneumonia, unspecified	485	Bronchopneumonia, organism unspecified
J18.1	Lobar pneumonia, unspecified		
J18.8	Other pneumonia, organism unspecified	486	Pneumonia, organism unspecified
J18.9	Pneumonia, unspecified		
A48.1	Legionnaires´s disease		
**Intraabdominal infections**			
K35.0-K35.8	Acute appendicitis	540.0-540.9	Acute appendicitis
K37	Unspecified appendicitis		
K65.0	Peritonitis	567.0-567.9	Peritonitis and retroperitoneal infections
K80.0	Calculus of gallbladder with acute cholecystitis	574.0	Calculus of gallbladder with acute cholecystitis
K80.3-K80.4	Calculus of bile duct with cholangitis	574.3	Calculus. of bile duct with acute cholecystitis
K81.0	Acute cholecystitis	575.0	Acute cholecystitis
K83.0	Cholangitis	576.1	Cholangitis
**Urinary tract infections**			
N10	Acute tubule-interstitial nephritis	590.0-590.9	Infections of kidney
N12	Tubulo-interstitial nephritis, not specified		
N39.0	Urinary tract infections, site not specified	599.0	Urinary tract infection, site not specified
**Central nervous system**			
A39.0-A39.9	Meningococcal infection	320.5	Meningococcal meningitis
G00.0	Haemophilus meningitis	320.0	Haemophilus meningitis
G00.1	Pneumococcal meningitis	320.1	Pneumococcal meningitis

G00.2	Streptococcal meningitis	320.2	Streptococcal meningitis
G00.3	Staphylococcal meningitis		
G00.8	Other bacterial meningitis	320.8	Meningitis due to other specified bacteria
G00.9	Bacterial meningitis, unspecified	320.9	Meningitis due to unspecified bacterium
G01	Meningitis in bacterial disease classified elsewhere	320.7	Meningitis in other bacterial diseases
G04.2	Bacterial meningoencephalitis and meningomyelitis		
**Skin- and soft tissue infections**			
A46	Erysipelas	035	Erysipelas
L03.1<br>L03.2<br>L03.3<br>L03.8<br>L03.9	Cellulitis and acute lymphangitis of other parts of limb<br>Cellulitis and acute lymphangitis of face and neck<br>Cellulitis and acute lymphangitis of trunk<br>Cellulitis and acute lymphangitis of other sites<br>Cellulitis and acute lymphangitis, unspecified		
M72.6	Necrotizing fasciitis	728.86	Necrotizing fasciitis
		729.4	Fasciitis unspecified
M00.0-M00.9	Pyogenic arthritis	711.0	Pyogenic arthritis
M01.3	Direct infections of joint in infectious and parasitic diseases classified elsewhere		
M86.0<br>M86.1<br>M86.2<br>M86.9	Acute haematogenous osteomyelitis<br>Other acute osteomyelitis<br>Subacute osteomyelitis<br>Osteomyelitis unspecified	730.0<br>730.2<br>730.9	Acute osteomyelitis<br>Unspecified osteomyelitis<br>Unspecified infection of bone
**Sepsis**			
A40.0-A40.9	Streptococcal sepsis	785.5	Shock
A41.0-A41.9	Other sepsis		
A48.3	Toxic shock syndrome		
R57.2	Septic shock		
B95.0-B95.8	Streptococcus and staphylococcus as the cause of diseases classified to other chapters.		
B96.0-B96.8	Other specified bacterial agents as the cause of diseases classified to other chapters		
**Endocarditis**			
I33.0	Acute and subacute infective endocarditis	421.0	Acute and subacute bacterial endocarditis
I33.9	Acute endocarditis, unspecified	421.1	Acute and subacute infective endocarditis in diseases classified elsewhere
I38	Endocarditis, valve unspecified	421.9	Acute endocarditis, unspecified
