# Supplementary material for: Incidence, recurring admissions and mortality of severe bacterial infections and sepsis over a 22-year period in the population-based HUNT study
Source: PLoS One. 2022 Jul 12;17(7):e0271263. doi: 10.1371/journal.pone.0271263 (PMC9275692; doi:10.1371/journal.pone.0271263)
Supplement: S2 Table — (PDF) [file pone.0271263.s002.pdf]

Supplementary table 2: Seasonal differences in incidence rates.

**Focus of infection**	**Incidence rate pr 100 000/year (with 95% CI)**	Incidence rate autumn/winter	Incidence rate spring/sommer
**Pneumonia**	639 (625-653)	341(331-352)	298 (289-308)
**UTI**	550 (537-563)	282 (273-291)	268 (259-277)
**Sepsis/bacteraemia**	334 (324-345)	151 (145-158)	144 (137-151)
**SSTI**	115 (109-121)	54.5 (50.5-58.7)	60.5 (56.3-65.0)
