# Supplementary material for: Incidence, recurring admissions and mortality of severe bacterial infections and sepsis over a 22-year period in the population-based HUNT study
Source: PLoS One. 2022 Jul 12;17(7):e0271263. doi: 10.1371/journal.pone.0271263 (PMC9275692; doi:10.1371/journal.pone.0271263)
Supplement: S3 Table — (PDF) [file pone.0271263.s003.pdf]

Supplementary table 3. Summary of results divided into eight different foci of infection, males.

Focus of infection	Total admissions (n)	First-time admissions (n)	Incidence rate pr 100 000/year (with 95% CI)	Proportion with recurrent infection (%, with 95% CI)	Proportion with positive blood culture (%, with 95% CI)	30-day mortality (%, with 95% CI)
**Pneumonia**	7,355	4,235	739 (717-761)	28.6 (27.3-30.0)	4.7 (4.1-5.4)	17.6 (16.5-18.7)
**UTI**	4,526	2,573	449 (431-467)	28.0 (26.3-29.8)	8.5 (7.5-9.6)	10.3 (9.1-11.5)
**Sepsis/bacteraemia**	3,411	2,002	350 (355-366)	18.9 (17.2-20.7)	40.9 (38.8-43.1)	15.3(13.8-16-9)
**IAI**	1,380	958	166 (157-178)	11.5 (9.6-13.7)	11.8 (9.9-14.0)	4.0 (2.9-5.4)
**SSTI**	1,037	692	121 (112-130)	15.2 (12.7-18.0)	4.4 (3.1-6.2)	3.3 (2.2-5.0)
**Bone/joint**	151	97	16.9 (13.9-20.7)	16.5 (10.3-25.3)	17.5 (11.1-26.5)	9.8 (4.9-16.9)
**Endocarditis**	152	63	11.0 (8.6-14.1)	15.9 (8.7-27.2)	38.1(26.9-50.7)	15.9 (8.7-2.7)
**CNS**	55	34	5.9 (4.2-8.3)	2.9 (0.4-18.7)	50.0 (33.5-66.5)	11.8 (4.4-27.9)
