# Supplementary material for: Incidence, recurring admissions and mortality of severe bacterial infections and sepsis over a 22-year period in the population-based HUNT study
Source: PLoS One. 2022 Jul 12;17(7):e0271263. doi: 10.1371/journal.pone.0271263 (PMC9275692; doi:10.1371/journal.pone.0271263)
Supplement: S4 Table — (PDF) [file pone.0271263.s004.pdf]

Supplementary table 4: Summary of results divided into eight different foci of infection, females

Focus of infection	Total admissions (n)	First-time admissions (n)	Incidence rate pr 100 000/year (with 95% CI)	Proportion with recurrent infection (%, with 95% CI)	Proportion with positive blood culture (%, with 95% CI)	30-day mortality (%, with 95% CI)
**Pneumonia**	5,855	3,713	554 (537-573)	25.5 (24.1-26.9)	5.2 (4.5-6.0)	13.4 (12.4-14.6)
**UTI**	6,895	4,266	637 (618-656)	26.6 (25.3-28.0)	6.8 (6.1-7.6)	7.4 (6.6-8.2)
**Sepsis/bacteraemia**	3,545	2,154	322 (309-336)	18.7 (17.1-20.3)	34.4 (32.4-36.4)	11.5(10.2-12.9)
**IAI**	1,524	1,050	156 (147-166)	11.1 (9.4-13.2)	8.0 (6.5-9.8)	3.3 (2.4-4.6)
**SSTI**	1,167	737	110 (102-118)	16.1 (13.7-19.0)	5.1 (3.8-6-9)	3.1 (2.1-4.7)
**Bone/joint**	96	76	11.3 (9.1-14.2)	7.9 (3.6-16.6)	21.1 (13.3-31.7)	3.9 (1.3-11.6)
**Endocarditis**	86	33	4.9 (3.5-6.9)	24.2 (12.5-41.8)	42.4 (26.8-59.7)	30.3 (17.0-50.0)
**CNS**	63	31	4.6 (3.3-6-6)	16.1 (6.8-33.8)	31.3 (17.5-49.3)	3.2 (0.4-20.3)
