# Supplementary material for: Incidence, recurring admissions and mortality of severe bacterial infections and sepsis over a 22-year period in the population-based HUNT study
Source: PLoS One. 2022 Jul 12;17(7):e0271263. doi: 10.1371/journal.pone.0271263 (PMC9275692; doi:10.1371/journal.pone.0271263)
Supplement: Attachment — Submitted filename: PLOS_Sever Bact Infection Norway 22 yr Cohort_Feb 2022.docx [file pone.0271263.s005.docx]

PONE-D-21-20080

Article Type: Research Article

Full Title: Incidence, recurring admissions and mortality of severe bacterial infections and sepsis over a 22-year period in the population-based HUNT Study.

This is an interesting well designed cohort study looking at population severe bacterial infection, with outcomes of determining specific infection site incidence rates, 30 days’ all-cause mortality, recurring admissions, positive blood culture. The cohorts were enrolled in 1995-1997 (70% acceptance rate) and 2006-2008– (54% acceptance rate). It would be of interest to determine if they were outcome differences between the two cohorts regarding severe infection over the years

Some minor comments

1. Was there any overlap in the HUNT 2 and 3 cohorts? What was retention like over the years.
2. The study was conducted in what can referred to as “a rural area”. However, urban areas may have various factors impacting on risks of infections and their outcomes, such as environmental factors – outdoor vs indoor lifestyles etc – should these be considered in the limitations.
3. Regarding the “implicit sepsis” related to organ dysfunction – could they have been an over diagnosis of sepsis in this group.
4. The low blood culture noted is an important finding – as clinicians often assume this is due to prior antibiotics. Was this possibility of prior outpatient antibiotic use explored?
5. About half the population got at least one infection needing admission – is this a true reflection of this Norwegian population– or due to higher cohort awareness? It is quite high especially as the age inclusion included young adults. In the questionnaire were participants advised to report to hospital for any suspected infection, or this is an expected figure in a none cohort population of a mixed age population from 20 yrs up upwards. What was effect of age on admissions?
6. The sex distribution would be of interest especially with UTI at different ages. This was common in young adults – were they mostly women?
7. Pre morbidities – did they play a factor in the incidence of infections. Fragility and low immunity in older population was discussed – but what of underlying illnesses?
8. Any idea what organisms were cultured in the different infections and age ranges – this would be useful information to guide clinical care.
9. The study was done in Norway – but most comparisons in discussion were with USA studies. Any related research in Europe – if so ow comparable are the findings?
10. Was there some temporal variations with the infections esp. related to weather changes.
11. Minor typos – Sepsis results section line167 UTI not UVI, and line 168 a not av.
12. Overall, the strength of this study is a large population cohort. Some biases to be considered could be changes within the health care systems over the years, as well as changes in health seeking behavior in a more informed cohort over time
