# Supplementary material for: Incidence, recurring admissions and mortality of severe bacterial infections and sepsis over a 22-year period in the population-based HUNT study
Source: PLoS One. 2022 Jul 12;17(7):e0271263. doi: 10.1371/journal.pone.0271263 (PMC9275692; doi:10.1371/journal.pone.0271263)
Supplement: Attachment — Submitted filename: Response to reviewers.docx [file pone.0271263.s007.docx]

**Comments from the reviewers:**

**Reviewer 1:**

*Authors have adequately addressed all the concerns from the first review appropriately. Some issues have been revised, some included in limitations while others re considered for potential follow-up work – which is acceptable.*

*One minor issue to consider is - whether patients with UTI died OF or died WITH UTI. This is more so since UTI was the second commonest cause of death following pneumonia. Were there any overlap infections at admission?
Much as one can clearly state some infections are a direct cause of death - I am not sure if one can say the same of UTI in most cases - as its common with very ill admitted patients esp. in the older population, - who may have another infection.
Comment 8 – regarding type of organisms need not be included in this manuscript.*

**Response:** Thank you for highlighting this important aspect, which will always be important to consider when reporting the all-cause 30-day mortality following any event. We agree that this is especially important when it comes to the urinary tract infections, and it should have been highlighted.

Of the 37298 participants having a hospital admission due to infection, 4628 had two infections codes and 918 had three or more. When reporting the 30-day all-cause mortality it is not possible to assess which of them contributed the most to death, and this is especially important to consider when it comes to the urinary tract infections. The manuscript has now been changed slightly on several places to make this important point clearer. (Lines 142-143, 160-162, 181, 188, 198-199 and 236-239).

**Reviewer 2:**

*I think the authors by and large have commented my questions and comments satisfactorily. It is, however, still a bit muddled with two cohorts and two follow-up periods although I have no doubt that incidence rates etc. have been computed correctly. I guess with the focus on age, the big differences between the two cohorts as regards median age (>10 y difference), death (28.9% vs. 3.9%), and follow-up time are okay.*

*There was one point that I think the authors have misunderstood (and admittedly, I did not explain it very clearly either!). They write: “Regarding competing risk analysis, this is a very interesting aspect, but we have chosen not to study different risk factors for infectious diseases in depth in this paper. We feel that competing risk analysis would have been more relevant if we had aimed at causal analysis between exposure and outcome.” When I referred to competing risk I did not think of different factors, but of death as competing risk to reinfection. That is, you can only be reinfected if you are alive. If e.g. a large proportion of a population dies shortly after the first infection, the denominator will change. It will actually change every time a person dies and it is therefore a good idea to incorporate a competing risk analysis, either using the Fine & Gray methods or treating death as censoring before time. Competing risk from death makes it hard to interpret your reinfection proportions. But again, as your study is mainly descriptive and not based on time-to-event regression analyses, I can live with that.*

**Response:** We acknowledge the reviewer’s comment on competing risk of death by other causes than the severe infections studied. To assess the amount of competing risk of death with our commonest group of infection, pneumonia, as an example, we assessed the proportion of participants who were censored because of death 1) before their first pneumonia and 2) before any recurrent pneumonia. The corresponding numbers are 13131 and 4066.

When studying a population that ages during follow-up, death will serve as a competing risk. In our study we descriptively reported the proportion who were subsequently reinfected, which we think serves the purpose of informing about the burden of reinfections on the population level. However, in the original analyses patients that did not survive their first infection were included in the denominator when calculating the proportion of recurrence. In accordance with the reviewer’s comment, this has now been changed, leading to slightly higher proportions of recurrence in all diagnosis groups. This has been changed in Table 2 on page 9 and changed and explained on lines 50, 119-120, 159, 179, 184 and 197. Figure 2 has also been changed accordingly. For future in depth investigations of reinfections, including analyses of risk factors for reinfection, we agree that time-to-event analyses accounting for competing risk is an appropriate tool.
